# Allelopathy and resource competition: the effects of *Phragmites australis* invasion in plant communities

**DOI:** 10.1186/s40529-017-0183-9

**Published:** 2017-06-29

**Authors:** Md Nazim Uddin, Randall William Robinson

**Affiliations:** 1 Department of Ecology & Environmental Management, College of Engineering & Science, Victoria University, Melbourne, VIC 8001 Australia; 2 Institute for Sustainability & Innovation, Victoria University, Melbourne, VIC 8001 Australia

**Keywords:** Allelopathy, Density-dependent phytotoxicity, Ecosystems, Invasion, *Phragmites australis*, Resource competition

## Abstract

**Background:**

*Phragmites australis*, a ubiquitous wetland plant, has been considered one of the most invasive species in the world. Allelopathy appears to be one of the invasion mechanisms, however, the effects could be masked by resource competition among target plants. The difficulty of distinguishing allelopathy from resource competition among plants has hindered investigations of the role of phytotoxic allelochemicals in plant communities. This has been addressed via experiments conducted in both the greenhouse and laboratory by growing associated plants, *Melaleuca ericifolia*, *Rumex conglomeratus*, and model plant, *Lactuca sativa* at varying densities with the allelopathic plant, *P. australis*, its litter and leachate of *P. australis* litter. This study investigated the potential interacting influences of allelopathy and resource competition on plant growth–density relationships.

**Results:**

In greenhouse, the root exudates mediated effects showed the strongest growth inhibition of *M. ericifolia* at high density whereas litter mediated results revealed increased growth at medium density treatments compared to low and high density. Again, laboratory experiments related to seed germination and seedling growth of *L. sativa* and *R. conglomeratus* exhibited phytotoxicity decreased showing positive growth as plant density increased and vice versa. Overall, the differential effects were observed among experiments but maximum individual plant biomass and some other positive effects on plant traits such as root and shoot length, chlorophyll content occurred at an intermediate density. This was attributed to the sharing of the available phytotoxin among plants at high densities which is compatible to density-dependent phytotoxicity model.

**Conclusions:**

The results demonstrated that plant–plant interference is the combined effect of allelopathy and resource competition with many other factors but this experimental design, target-neighbor mixed-culture in combination of plant grown at varying densities with varying level of phytotoxins, mono-culture, can successfully separate allelopathic effects from competition.

**Electronic supplementary material:**

The online version of this article (doi:10.1186/s40529-017-0183-9) contains supplementary material, which is available to authorized users.

## Background

Allelopathic interference by invasive plant species has potential to impact seed germination, seedling growth, development and establishment of neighbouring plant species, as well as of the same species, in both natural and agricultural systems (Bich and Kato-Noguchi 2014; Dorning and Cipollini 2006; Lara-Núñez et al. 2006). Allelopathy has been considered an important attribute to the success of an invasive species in natural ecosystems (Callaway and Ridenour 2004; Kimura et al. 2015; Lorenzo et al. 2010). The sources of allelochemicals released into the rhizosphere include leaching from leaves and other aerial parts, volatilization, root exudation and litter decomposition (Hussain and Reigosa 2012; Uddin et al. 2012; Weir et al. 2004).

*Phragmites australis*, a ubiquitous wetland plant, is considered one of the most invasive species in the world (Uddin et al. 2012) however, the origin of the species is still unclear (Plut et al. 2011). A perennial graminaceous plant, to 3 m tall, it reproduces mainly through rhizomes and, at low frequency, through seeds. *P. australis* grows in all temperate zones of the world, especially North America, most countries in Europe, some parts of Canada and Australia (Hocking et al. 1983; Kulmatiski et al. 2011), being especially common in south-eastern Australia (Kettenring et al. 2011; Morris et al. 2008). The worldwide, regional and local distribution and abundance of *P. australis* has expanded over the last 150 years and in most areas it forms dense monocultures (Saltonstall and Miller 2005). Due to the impacts of *P. australis* invasions, habitats have been diminished or altered significantly for other flora and fauna causing loss of biodiversity and ecosystem functions (Mack et al. 2000). Several studies have identified chemicals within *P. australis* organs which have antialgal, antifungal or antibacterial effects (Li and Hu 2005). Previous allelopathic studies have shown that water extracts, decomposed materials, root exudates and specific identified chemicals of *P. australis* organs have strong phytotoxic effects on germination, growth, and establishment of other plant species (Kettenring et al. 2011; Rudrappa et al. 2007, 2009; Uddin et al. 2012, 2014a, b, c) and thus, it is assumed that *P. australis* achieves its competitive advantages over invasion process into wetlands through allelopathy (Bains et al. 2009; Rudrappa et al. 2007).

While *P. australis* has clearly shown phytotoxic potential, the effects should be considered in more ecologically realistic ways by differentiating allelopathic interactions from resource competition. The allelopathic effects might be masked by resource competition among target plants (Barto and Cipollini 2009; Weidenhamer et al. 1989). A better understanding of dose–response relationships of allelochemicals would help to clarify this issue. Toxin dilution is thought to occur because plants share and compete not only for resources but also for toxin (Hansi et al. 2014; Hoffman and Lavy 1978; Suman et al. 2002). Population density modifies phytotoxic effects through dilution of available toxins among plants (i.e. phytotoxicity decreases as plant density increases) (Thijs et al. 1994). Thus, the dose of a phytotoxin received by a plant is inversely related to plant density. This toxin dilution study can be performed using density-dependent experiments, and may be a potential tool for exploring the effects of phyto-toxins on plant growth as well as for differentiating the resource competition from allelopathy. As the study of allelopathic interactions may be hindered by the lack of proper experimental methods, it may be more productive to first demonstrate explicit interference, by allelochemicals rather than rely solely on explanations that involve resource competition or other mechanisms (Barto and Cipollini 2009; Thijs et al. 1994).

Although the use of activated carbon (AC) as a soil amendment, has use in determining distinct differences between allelopathy and resource competition (Inderjit and Callaway 2003), AC may change the availability of soil nutrients (Weißhuhn and Prati 2009). Weidenhamer (2006) proposed allelopathic effects might be differentiated experimentally using the density-dependent nature of phytotoxic effects, in turn, causing deviations from predicted growth–density relationships. The effects by allelochemicals depends on density of neighbour-target plant species and might be masked by resource competition at high density (Weidenhamer 1996). Density-dependent models suggest that yield decreases with increasing density due to resource competition acting as a dominating factor. Alternatively, results due to allelopathy show a slow decrease of yield or even increase in yield as density increases, until density reaches a point where resource competition among neighbouring target plant becomes the dominating factor. The yields of exposed plants to pure chemicals (Andersen 1981), ground tissue of allelopathic plants (Tseng et al. 2003) and soil mediated allelopathic plants (Weidenhamer et al. 1989) are consistent with the assumptions of a density-dependent phytotoxicity model. Andersen (1981) found that reduced plant seedlings of soybeans may partially reduce the negative effects of herbicides as well as Weidenhamer et al. (1989) and Tseng et al. (2003) stated that phytotoxicity decreased as plant density increased.

Therefore, this study has been designed to determine the occurrence and magnitude of potential allelopathic effects mediated by *P. australis* root exudates, its litter and extracts of litter with a wide range of doses through a density-dependent approach. This method might be effective in distinguishing the allelopathic interactions of *P. australis* with neighbouring plant species from resource competition. We hypothesized that phytotoxic effects of allelochemicals depend on the neighbouring plant density, due to phytotoxins dilution among individual plants.

## Methods

### Study site, plant litter and soil sample collection

Fallen leaves of *P. australis* were collected in June 2011 from natural stands adjacent to Cherry lake (37°51′ 30″S, 144°50′ 5″E), a coastal wetland in Altona, Melbourne, Australia. All samples were placed into sealable plastic bags for transportation to the laboratory. Plant samples were sorted from other plant residue and debris, then kept at room temperature to air dry until constant dry weight. After desiccation, sorted samples were cut into small pieces (<2 cm) and preserved in plastic ziplock bags until use. Soil samples were collected from the top layer of *P. australis* free areas of the same study site, separated from other organic materials, dried at room temperature and kept in ziplock bags after passing through a 2 mm sieve.

### Choice of target species

Seeds of several species were used to determine any differential response in native, introduced and model species to allelopathy. Seed capsules of the native species, *Melaleuca ericifolia* and introduced species, *Rumex conglomeratus* were collected in May 2011 from Cherry Lake and stored in paper bags at room temperature for 1 week. Seeds were shaken from the capsules and sieved to remove empty capsules and other detritus. Associated species were used because of ecological relevance. Seeds of a model species, *Lactuca sativa* were purchased from a commercial source (DT Brown Seeds, South Windsor, NSW, Australia). Lettuce was selected as it is widely used in phyto-toxicity bioassays. Model species is easily grown, minimizing the risk of observed growth differences, due to factors other than treatments applied in the experiments.

### Greenhouse experiments

#### Root exudates mediated effects on *M. ericifolia* seedlings

Spring buds of *P. australis* with rhizome attached were collected on 8 September 2011 from Cherry Lake. Each live rhizome was cut to contain exactly one active node, weighed and planted within 6 h of collection in 7 L plastic pots lined with watertight plastic bags filled with 4 L substrate [a 1:7 mixture of unsterilized river sand and potting mix soil respectively (Earth-wise Growing Essential, Australian Prime Fibre Pty Ltd, Queensland-4660)] and 20 g inoculum collected from *P. australis* field. Potting mix contained organic materials (pine bark), living organisms (bacteria, fungi and protozoa), minerals, fertilizers additives and more in details in our previous studies (Uddin et al. 2014b). The control pots containing substrates only were kept with other treated pots in the same greenhouse conditions until *M. ericifolia* juvenile plants were replanted. To all of the pots, 1 g L^−1^ of mixed pelletised fertilizer (Pivot fertilizer-900; N-P-K: 16-8-9) was incorporated into the tilled topsoil bimonthly. Pots were kept in a naturally-lit greenhouse at 23 ± 3 and 12 ± 2 °C day/night temperature and watered regularly with an auto irrigation system equipped by micro sprinklers at the soil surface to keep soil moist at a level of 55 ± 5%, similar to field soil. Soil moisture was monitored weekly by measuring water content in soil randomly collected from the pots. Pots were randomly shuffled every week to minimise the spatial effects and unwanted germinant (weeds) were removed. Tube stock of *M. ericifolia* (6 months old), grown in potting mix, were purchased from ‘Go Native Landscapes Pty Ltd’ (Inverloch, Victoria, 3996, Australia) grown from seeds collected from *M. ericifolia* stands at Dowd Morass wetland in Inverloch, Victoria, Australia. On 10 November 2011, the purchased *M. ericifolia* juvenile plants were planted into the prepared pots with and without *P. australis* by three neighbour densities (one, two, and four plants per pot) with three replicates of each treatment. After 6 months of *M. ericifolia* growth, all plants (*P. australis* and *M. ericifolia*) were harvested and data collected on above-ground biomass (AGB), below-ground biomass (BGB), root–shoot length, plant height, stem diameter, and number of growth points (terminal branches).

#### Litter mediated effects on *M. ericifolia* seedlings

*Phragmites australis* free soil (200 g) was set in 1.5 L pot, moistened to approximately field saturation level and kept for 2 days in the greenhouse. Equal size 2-month old *M. ericifolia* seedlings grown in naturally-lit greenhouse were then replanted in each pot with a density of one, two, and four plants at eight replicates. After 3 weeks of plant acclimation, equal size litter (≤2 cm) was put on soil surface in the pot with a concentration of 4 g/100 g of soil, but no litter for control and kept as per the above conditions. The amount of litter present on the soil surface was identified on previous field observations to be the amount of litter fall in a 1-m^2^ quadrate. After 4 months, the survived plants with mixed replicates **(**low density = 8, medium density = 3 and high density = 4) were harvested and measured for the various phenotypic characteristics.

### Laboratory experiments

#### Litter leachate mediated effects on *R. conglomeratus* seedlings

Litter leachate was made by soaking litter in distilled water with a concentration of 10% (100 g litter in 1 L water). Left to soak for 24 h, the leachate was filtered with cheese-cloth, centrifuged at 3000 rpm and filtrate then used for the experiment with pH adjustment at 6.5 with 1 N NaOH and 1 N HCl to avoid non-relevant effects of leachate due to pH. Fifty grams of *P. australis* free soil was moistened with 30 mL litter leachate at four different concentrations (0, 2.5, 5.0, and 10.0%) in 250 mL container, and incubated for 24 h. In general, the most of the bioassays are conducted in the allelopathy study at concentrations of 1–5% (the weight or mass of the plant matter per volume of solvent) (Reigosa et al. 2013). Therefore, we used a wide range of doses to ensure encompassing lowest dose for an observable effect, as well as the highest dose for maximal effect (Belz et al. 2007). Equal numbers (15 seeds with three replicates) of *R. conglomeratus* seeds were sown in each pot to ensure emergence of adequate numbers of even-age seedlings. All pots were then placed in a growth chamber (Westinghouse, Electrolux home products, Australia) set to 25/15 °C day/night temperature and a 12 h photoperiod with illumination of 84 μmol s^−1^ m^−2^. One week after emergence, seedlings were thinned to a density of one, two, four, and eight in each pot. Water loss by evaporation was measured by weight and compensated with leachate twice in a week. Plants were harvested and measured the phenotypic and physiological characteristics after 6 weeks of treatments.

#### Unburnt versus burnt litter extracts mediated effects on *L. sativa* seedlings

*Phragmites australis* litter was placed in a furnace at 300 °C for 1 h to produce burnt litter and a phyto-toxicity test was conducted with extracts of both type of litter (unburnt and burnt). Extracts were made by mixing unburnt and burnt litter powder passed through 0.5 mm sieve with distilled water to a concentration of 10%. After a 24 h period, it was filtered with cheese cloth and centrifuged at 3000 rpm. The resultant filtrate then used as the extract for the experiment with pH adjustment at 6.5 with 1 N NaOH and 1 N HCl, to avoid non-relevant effects of extracts due to pH. Extract (5 mL) at four different concentrations (0, 2.5, 5.0, and 10.0%) was placed into a sterile 9 cm Petri dish containing two sterile sheets of filter paper (Whatman No. 1). At least, three replicates were used for each treatment with density of one, two, four and eight pre-germinated *L. sativa* seedlings. Petri dishes were sealed with parafilm (Pechiney, Plastic Packaging Company, Menasha, WI 54952) then placed in polyethylene bags to prevent water loss by evaporation and to avoid contamination by fungi and bacteria. The prepared dishes were arranged in a completely randomized design (CRD) and placed in a growth chamber according to above mentioned conditions. The Petri dishes were randomized each day to minimize the spatial effect. After 7 days of experiment, phenotypic characteristics of the grown of the plants were measured.

#### Litter mediated effects on *L. sativa* seed germination

Seed germination bioassay was conducted using the ‘sandwich’ method adopted from Fujii et al. (2004). Four different concentrations of air dried litter by weight (0, 2.5, 5.0, and 10%) were placed in between two layers of 0.5% agarose (total 10 mL) in a container of 4.5 by 5.5 cm. Agarose was autoclaved at 121 °C for 15 min and subsequently cooled at room temperature. Agar media was used due to higher bio-availability and greater exposer of the allelochemicals to the receiver plants through diffusion (Duke 2015). In addition, microbial community in substrate convert phytotoxins to more active or less active compounds but autoclaving may eliminate the possibility (Duke et al. 2009). Using autoclaved agarose with litter, the seeds of *L. sativa* at a density of four, eight, and 16 were placed on each container and incubated for 7 days as above condition with three replicates. The germination and biometric parameters were measured at the end of the experiment.

### Phenolics determination

Total phenolics (TP) and water soluble phenolic (WSP) content were measured in unburnt and burnt litter with three replicates, due to a causal relationship between phenolic content and phytotoxicity in plant (An et al. 2001; Huang et al. 2003). Approximately 100 mg portion of powder (burnt and unburnt litter) was weighed out and transferred to Eppendorf tube. After addition of 5 mL of 70% acetone for TP and distilled water for WSP, these were incubated at 4 °C for 1 h to extract phenolics followed by centrifuging at 15,000 rpm for 10 min at 4 °C. The 0.5 mL of the supernatant was taken and made up to 1 mL with distilled water. Then 5 mL of 2% Na_2_CO_3_ in 0.1 N NaOH was added and mixed using vortex mixer (Vortex Mixer, VOU1, Ratek Instruments Pty Ltd., Australia). To the mixture obtained by the above process 0.5 mL Folin-Ciocalteu reagent was added and mixed. After 2 h, absorbance was read at 760 nm. Based on the standard curve, phenolic was determined as gallic acid equivalents of the sample (mg TP and WSP per g sample).

### Chlorophyll measurement

Photosynthesis is the basic physiological process of plant growth and it has been inhibited by allelochemicals through influencing the chlorophyll content of the exposed plants (Qian et al. 2009; Zhou and Yu 2006). To gain an understanding of the physiological mechanism influenced by allelochemicals, chlorophyll *a* (Chl *a*), chlorophyll *b* (Chl *b*) and total chlorophyll were determined. Approximately 15 mg of fresh leaf of affected *R. conglomeratus* was placed in 7 mL of *N*–*N* dimethylformamide (DMF) for 24 h in darkness at 4 °C for extraction (Moran and Porath 1980). The absorbance of the extracts was measured spectrophotometrically at 664 and 647 nm and chlorophyll was determined following the equation proposed by Inskeep and Bloom (1985).

### Statistical analyses

All the experiments were conducted in a completely randomized design with at least three replicates. All data were referred as percentage of the values of control treatments at each density and analysed using IBM SPSS statistics 21.0 and Microsoft Excel 2010. Two-way ANOVA was used to show the effects of *P. australis* litter leachate on seedling densities of *R. conglomeratus*. Three-way ANOVA was designed to perform the effects of different materials (unburnt and burnt) with different concentrations of *P. australis* litter extract on growth of *L. sativa* seedling densities. Again, using two-way ANOVA we measured the effects of different concentrations of *P. australis* litter mediated agarose on germination and growth of *L. sativa* with different seed densities. In all cases, variance homogeneity was tested using Levenes’s test and transformed if necessary (square root). Significance tests were performed using univariate analysis of variance with 2-sided Duncan’s tests at the 0.05 probability level. Furthermore, the competitive effect of a neighbour species on a target species was quantified through linear regression procedures. The slope of the regression and the correlation coefficient that evaluates the dependence of each measured variables of exposed plants such as biomass (above-ground, below-ground and total biomass) and root length on the concentration of the residues and residue extracts as well as plant and seed density, were calculated. Again, an effort has been made to determine whether the difference in slopes between treatment and control was statistically significant by Real Statistics Resource Pack Software plug-in Microsoft Excel 2010 with log-transformed data (Zaiontz 2014). Slopes were compared via the slopes test [(=SlopesTest(x1, y1, x2, y2)] and the T-distribution [(=TDIST(x, df, tails)] following the method for comparing slopes of two independent samples developed by Zaiontz (2014).

## Results

### Greenhouse experiments

Root exudates of *P. australis* affected the biomass and other morphological characters of *M. ericifolia* at various densities (Table 1). Most of the growth parameters decreased with increasing plant density indicating that plant density was the most significant factor for influencing the growth of *M. ericifolia* such as AGB (F_2, 6_ = 26.96, *P* < 0.01), BGB (F_2, 6_ = 52.91, *P* < 0.001), total biomass (F_2, 6_ = 29.04, *P* < 0.001), root length (F_2, 6_ = 39.95, *P* < 0.001), plant height (F_2, 6_ = 14.65, *P* < 0.005), and growth points (F_2, 6_ = 111.83, *P* < 0.001). The slopes of all the growth parameters like AGB (*t* = 2.24), BGB (*t* = 3.12), total biomass (*t* = 2.90), root length (*t* = 2.82), plant height (*t* = 2.92), and growth points (*t* = 2.90) were significant compared to control with degree of freedom *df* 14 and significance level *α* 0.05.

**Table 1:** Changes of biomass and morphological characters at various density of *Melaleuca ericifolia* in treatments of *Phragmites australis* root exudates and litter as % of control

Plant density/pot	Source of materials	Aboveground mass	Belowground mass	Total biomass	Root length	Plant height	Growth point
Low	Root exudates	94.47 ± 3.09 a	88.03 ± 6.16 a	93.87 ± 2.54 a	100.00 ± 7.22 a	90.56 ± 3.09 a	98.27 ± 5.38 a
	Plant litter	6.18 ± 0.84 a	7.06 ± 1.13 a	6.35 ± 0.86 a	34.02 ± 2.71 a	55.93 ± 4.08 a	11.38 ± 1.47 a
Medium	Root exudates	62.98 ± 4.26 a	67.90 ± 10.55 a	63.42 ± 4.77 a	85.86 ± 7.89 a	93.74 ± 8.28 a	71.72 ± 8.27 b
	Plant litter	22.85 ± 4.40 b	25.15 ± 4.80 b	23.27 ± 4.26 b	61.75 ± 3.49 b	82.74 ± 1.49 b	17.94 ± 2.18 b
High	Root exudates	165.45 ± 16.70 b	273.33 ± 24.04 b	171.65 ± 17.10 b	195.56 ± 12.37 b	126.92 ± 2.21 b	194.42 ± 3.80 c
	Plant litter	10.81 ± 1.24 a	10.02 ± 2.15 a	10.63 ± 1.35 c	66.50 ± 8.80 b	66.51 ± 6.42 ab	8.22 ± 0.71 a

Average (±standard error) values in the column followed by same letter are not significantly different at the 0.05 level by Duncan’s test

Treated soil mixed with or without the leaf litter of *P. australis* reduced growth of *M. ericifolia* by inhibiting biomass, root length, plant height, stem diameter and number of growth points at various densities (Table 1). A differential in reduction was observed in the whole experiment but medium density treatments had increased growth compared to low and high density treatments. Total dry biomass per plant was reduced by 94, 77 and 90% at low, medium and high density respectively. The reduction of root length (65%), plant height (44%), stem diameter (61%) and number of growth points (89%) was more severe at low density than high density, but comparatively less inhibited at medium density. Growth parameters like AGB (F_2, 12_ = 20.90, *P* < 0.001), BGB (F_2, 12_ = 16.39, *P* < 0.001), total biomass (F_2, 12_ = 20.71, *P* < 0.001), root length (F_2, 12_ = 18.22, *P* < 0.001), plant height (F_2, 12_ = 6.65, *P* < 0.01), stem diameter (F_2, 12_ = 28.83, *P* < 0.001), and number of growth points (F_2, 12_ = 6.41, *P* < 0.01) varied significantly across the densities. Regression analyses among densities and growth parameters of *M. ericifolia* showed that the difference in slopes in the pots to *P. australis* treatments compared to the control were not significant such as AGB (*t* = 1.96), BGB (*t* = 1.70), total biomass (*t* = 1.87), plant height (*t* = 1.97), and number of growth points (*t* = 0.26) except root length (*t* = 4.29) with *df* 24 and *α* 0.05.

### Laboratory experiments

**Fig. 1:**
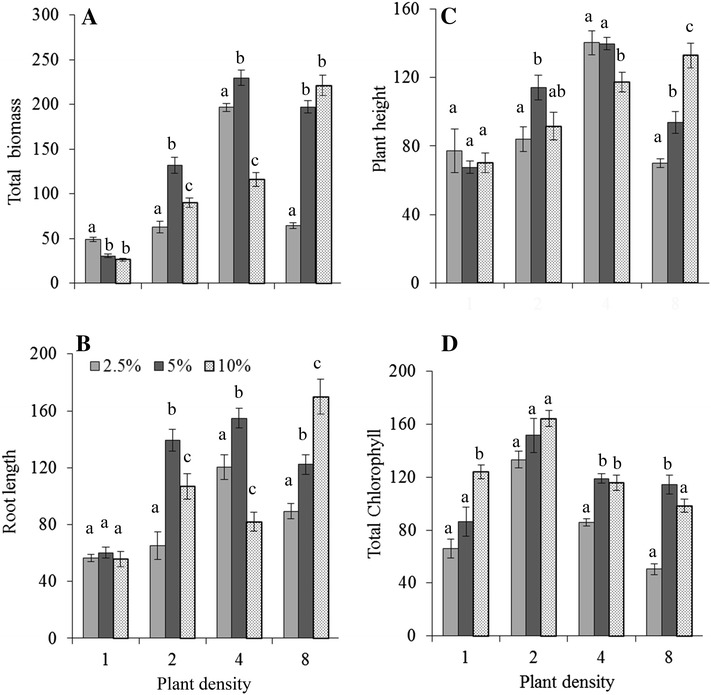
Effects of *Phragmites australis* litter leachate on **A** total biomass, **B** root length, **C** plant height and **D** total chlorophyll of *Rumex conglomeratus* seedlings at different densities. Values (weight per plant) are means as % of control treatments at each density ± standard errors (*n* = *3*). *Letters* indicate homogenous subgroups (*P* ≤ 0.05) at each density in Duncan’s test

Plants grown in *P. australis* litter leachate treated soil showed that the growth, biomass and other morphological and physiological parameters of *R. conglomeratus* were affected by both leachate concentration and density of test plant (Fig. 1; Table 2; Additional file 1: Figure S1). The degree of inhibition and stimulation by leachate was density-dependent. Generally, all growth parameters (AGB, BGB, total biomass, root length, plant height, number of leaves and chlorophyll content) increased with increasing plant density at lower and medium leachate concentration, but there were occurrences at a higher level. The highest biomass reduction was observed at the low plant density (one seedling/pot) treatment by 51 at 2.5% extract amended soil compared to control soil whereas 49 and 35% were in medium (two seedlings/pot) and high (eight seedlings/pot) density respectively but stimulated at intermediate (four seedlings/pot) density by 49%. All results showed a reduced leachate effect in relation to plant density and there was a clear trend to stimulation of growth as density increases. The variation in the magnitude of stimulation with density demonstrates dose-dependency of the stimulatory response, whereas no stimulatory effects were observed in the control. Deviations in the relationships between plant growth parameters and density were pronounced, however, comparisons of the regression lines showed that the differences in observed slopes were not significant with exception of root length (*t* = 2.92) at 2.5% leachate, AGB (*t* = 6.54), BGB (*t* = 2.38), total biomass (*t* = 4.19) and root length (*t* = 2.97) at 5.0% leachate whereas at 10.0% leachate most of the slopes were significant except number of leaf (*t* = 1.47) and total chlorophyll (*t* = 1.54) with *df* 20 and *α* 0.05.

**Table 2:** Results of two-way ANOVA (*F*-ratios) showing the effects of *Phragmites australis* litter leachate (C) on seedling densities (D) of *Rumex conglomeratus*

Factor	*df*	*F* ratio and probability						
		Above-ground biomass	Below-ground biomass	Total biomass	Root length	Plant height	Number of leaf/plant	Total chlorophyll
Concentration (C)	2	68.15***	40.02***	71.26***	23.49***	3.11^ns^	9.00***	40.39***
Density (D)	3	316.71***	267.80***	313.54***	51.72***	39.01***	5.50**	49.22***
C × D	6	74.66***	28.92***	70.88***	17.87***	9.12***	5.72***	3.99**
Error	24							
Total	36							

Data used here as % of control. Level of significance: ***, **, * and ^ns^ indicate significant difference at *P* ≤ 0.001, *P* ≤ 0.01, *P* ≤ 0.05 and non-significant respectively

**Fig. 2:**
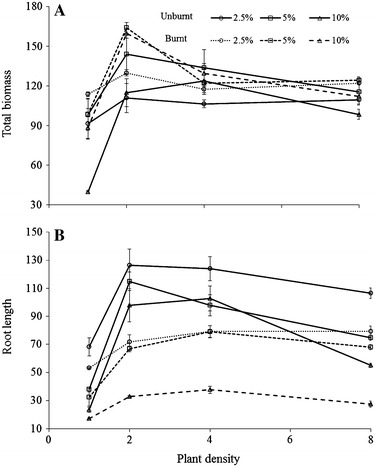
Relationship of **A** total biomass and **B** root length with seedling densities of *Lactuca sativa* grown at different concentrations of *Phragmites australis* litter (unburnt versus burnt) extracts. Values (weight per plant and length per plant) are means as % of control treatments at each density ± standard errors (*n* = *3*)

**Fig. 3:**
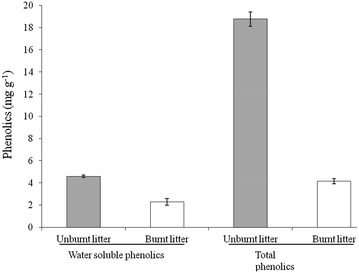
Total phenolics (TP) and water soluble phenolics (WSP) *Phragmites australis* litter (unburnt versus burnt). Values are means ± standard errors (*n* = *3*)

The effects of unburnt and burnt litter extracts of *P. australis* on the growth of *L. sativa* seedlings indicated significant reduction in terms of AGB, BGB, total biomass, root and shoot length (Table 3; Fig. 2; Additional file 1: Figure S2). Greater inhibition was observed with increasing concentration of the aqueous extracts. Materials (unburnt versus burnt), concentrations and densities all showed differential effects individually and interactively on biomass and root length of *L. sativa* (Table 3). BGB was inhibited more than AGB, but AGB–BGB ratio showed significant individual and interactive effects (Table 3). Inhibition was higher in unburnt residue extract, than burnt extracts (Fig. 2; Additional file 1: Figure S2) but the degree and magnitude of inhibition on AGB, BGB, total biomass and root length was not strongly correlated with the TP and WSP of the extracts. TP in unburnt versus burnt was 17.77 ± 0.64 versus 4.15 ± 0.24 mg g^−1^ whereas WSP was 4.59 ± 0.1 versus 2.3 ± 0.3 mg g^−1^ (Fig. 3). Although phenolics were significantly lower in burnt residue extracts, the inhibition shown by burnt extracts was not significantly lower than unburnt residue (Fig. 2; Additional file 1: Figure S2, available as additional material). The observed deviations (inhibitory and stimulatory) from the growth–density relationship were measured by the comparison of the slopes among treatments. The slope difference was significant at 10% unburnt extract in AGB (*t* = 3.90), BGB (*t* = 3.85) and total biomass (*t* = 4.51), but BGB was highly affected even at 2.5% (*t* = 3.12) and 5.0% extract (*t* = 2.67) with *df* 20 and *α* 0.05. Whereas, only significant slope difference was observed by burnt extract on BGB in each extract concentration such as 2.5% (*t* = 3.44), 5.0% (*t* = 3.61) and 10.0% (*t* = 4.57), with *df* 20 and *α* 0.05. Medium density had larger biomass and root length compared to low and higher density (Fig. 2; Additional file 1: Figure S2, available as additional material). Total biomass of *L. sativa* was significantly higher in burnt extracts than unburnt (Fig. 2). The total biomass increased with increasing density as compared to the control, except for density one in both unburnt and burnt litter extract.

**Table 3:** Results of three-way ANOVA (*F*-ratios) showing the effects of different materials (unburnt and burnt) (M) with different concentrations (C) of *Phragmites australis* litter extract on growth of *Lactuca sativa* seedling densities (D)

Factor	*df*	*F* ratio and probability				
		Above-ground biomass (AGB)	Below-ground biomass (BGB)	Total biomass	AGB–BGB ratio	Root length
Materials (M)	1	64.76***	1.92^ns^	25.55***	64.27***	246.88***
Concentration (C)	2	10.54***	50.81***	9.71***	93.17***	180.34***
Density (D)	3	37.02***	23.85***	40.55***	5.20**	154.91***
M × C	2	11.03***	4.44*	4.55*	27.20***	19.55***
M × D	3	7.41***	1.57^ns^	3.41*	11.98***	16.25***
C × D	6	6.91***	3.78**	6.10***	2.06^ns^	6.19***
M × C × D	6	1.00^ns^	1.34^ns^	1.01^ns^	2.96*	2.29*
Error	48					
Total	72					

Data used as % of control. Level of significance: ***, **, * and ^ns^ indicate significant difference at *P* ≤ 0.001, *P* ≤ 0.01, *P* ≤ 0.05 and non-significant respectively

**Fig. 4:**
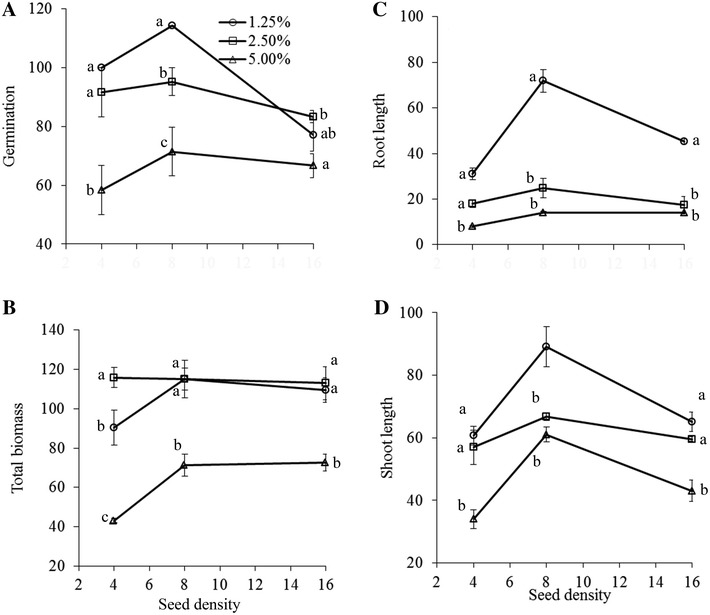
**A** Germination percentage, **B** total mass, **C** root length, and **D** shoot length in different concentrations of *Phragmites australis* litter mediated agarose at different seed densities of *Lactuca sativa*. Values (per plant) are means as % of control treatments at each density ± standard errors (*n* = *3*). *Letters* indicate homogenous subgroups (*P* ≤ 0.05) at each density in Duncan’s test

Germination bioassay showed differential effects on germination and growth parameters (Table 4; Fig. 4; Additional file 1: Figure S3). Germination percentage and AGB were significantly affected by both interactive and individual effect of residue concentration and density. All other measured parameters (BGB, total biomass, root and shoot length) varied significantly across the residue concentrations and density individually but not interactively (Table 4). BGB was the lowest at lower density and decreased (13, 31 and 68%) with increasing concentration (1.25, 2.5 and 5.0% respectively) compared to control while highest at the intermediate density. Root and shoot length was always higher along low concentrations at each density but they were significantly higher at medium densities (Fig. 4C, D). Again, these lowered with increasing and decreasing densities (Fig. 4). The difference in slopes was significant in AGB (*t* = 3.55), BGB (*t* = 3.56), total biomass (*t* = 4.14) and root length (*t* = 3.17) only at 5.0% concentration with *df* 14 and *α* 0.05.

**Table 4:** Results of two-way ANOVA (*F*-ratios) showing the effects of different concentrations (C) of *Phragmites australis* litter mediated agarose on germination and growth of *Lactuca sativa* with different seed densities (D)

Factor	*df*	*F* ratio and probability					
		Germination percentage	Above ground biomass	Below ground biomass	Total biomass	Root length	Root length
Concentration (C)	2	24.17***	71.35***	30.54***	53.59***	91.53***	29.67***
Density (D)	2	7.61***	7.95**	7.19**	6.19**	14.99***	20.82***
C × D	4	2.76*	4.51*	0.43^ns^	2.05^ns^	2.40^ns^	1.95^ns^
Error	18						
Total	27						

Data used as % of control. Level of significance: ***, **, * and ^ns^ indicate significant difference at *P* ≤ 0.001, *P* ≤ 0.01, *P* ≤ 0.05 and non-significant respectively

## Discussion

Analysis of growth–density relationships is useful tool for understanding the resource competition and allelopathic interference between plants of the suspected invasive species (Weidenhamer et al. 1989). The issue ‘separating allelopathy from resource competition’ is controversial in natural ecosystems (Inderjit and del Moral 1997; Weidenhamer 2006) but it is important in plant–plant interactions to evaluate the relative contribution and identify the mechanisms involved in their biological invasion processes. The replacement series design (Dekker et al. 1983) and using activated carbon (Ridenour and Callaway 2001) have been proposed that could yield insights into the nature of plant–plant interactions, and provide evidence for allelopathy. However, both methods have some complications in allelopathy studies (Goldberg and Werner 1983; Goldberg and Fleetwood 1987; Lau et al. 2008) whereas density-dependent phytotoxicity test is able to demonstrate in differentiating the relative contributions of those mechanisms in plant–plant interactions (Weidenhamer et al. 1989).

In general, the yield (in terms of growth and development) of plants decreases with increasing density; in contrast, density-dependent phytotoxicity studies reveal that plant growth may be positively influenced up to the point where resource competition acts as the dominant factor. Density-dependent phytotoxicity studies imply a positive feedback between population density and phytotoxins present in a system, as the toxin is shared among increased plant biomass with each plant receiving a proportionately smaller amount of toxin. Density-dependent phytotoxicity stands in contrast to resource competition as increased growth of plants at low density is dependent on large part to the amount of resources available. Despite the allelopathic potential of *P. australis* on associated and model plant species, as shown by the growth of *M. ericifolia*, *R. conglomeratus* and *L. sativa* observed in this study was masked by the resource competition but the allelopathic effects of *P. australis* are well supported (Rudrappa et al. 2007; Uddin et al. 2012, 2014a, b, c). These studies showed that water extracts of different organs, residue decomposition and root secreted phytotoxins had negative effect on germination, growth, and development of other plant species.

In greenhouse experiments of this study, the strongest growth inhibition was observed in high density treatments when compared to low and medium density. This demonstrates resource competition is the dominating factor, consistent with other studies (Inderjit and del Moral 1997; Uddin et al. 2014b). Our previous studies demonstrated that allelopathy through root exudates of *P. australis* had relatively low contribution in suppression of *M. ericifolia* in comparison to other competitive effects. Again the *P. australis* litter mediated soil experiment showed the highest root suppression of *M. ericifolia* potentially due to allelochemicals leached from litter mediated soil as the intermediate density of *M. ericifolia* showed increased growth compared to low and high density. The findings are well supported by the total assumptions of density-dependent phytotoxicity concept proposed by Weidenhamer (2008). This study states that growth is reduced at low but diminished at high density compared to control; and plant growth is highest at intermediate density, due to a reversal in slope of the predicted growth–density line.

In addition, the laboratory experiments showed a clear density-dependent phytotoxic effect, a result well aligned with other studies (Hansi et al. 2014; Lambertini et al. 2012) where allelochemicals, herbicides and inorganic compounds such as copper showing phytotoxicity is density-dependent. Our results suggest the relationship between growths, in terms of biomass, root length, plant height and plant density of *R. conglomeratus* exhibits a reversal in slope indicating the presence of phytotoxins in the litter leachate mediated soil used in the bioassay. The significant variation in phenolic content of unburnt versus burnt residues did not reflect the associate effects on plant growth suggesting heat induced transformation of phenolic compounds might be effective in suppression of plant growth, even though these residues may contain small amount of phenolic compounds. This result is well aligned with the study of Zhang et al. (2012) who found that there was no significant difference between unburnt versus burnt residues of *Flaveria bidentis* (L.) Kuntze on the growth of wheat (*Triticum aestivum* L.) seedlings. However, our studies found higher inhibitory effects in unburnt than burnt residue extract but it was not a true reflection of causal relationships between total phenolics and growth variables. Seed germination study showed that germination percentage and root–shoot length of *L. sativa* are a function of both concentration and the amount of phytotoxin available per seed. The inhibition increased as the concentration increased at lower density but stimulation was observed with intermediate density in most of the cases. This suggests that lower seed density increases the availability of phytotoxin per seed. Weidenhamer et al. (1987) found that even lower phytotoxin concentration may cause similar or greater inhibitory effects than higher concentrations, when the amount of phytotoxin per seed is greater.

In general, allelopathy research is more concerned with using concentrations, the introduction of soil microbes, and an autotoxicity test, involving a wide range of associated plant species in the bioassays, by questioning whether those involved are ecologically relevant. Despite this, it may be difficult to determine with some precision as to those occurring naturally in the field, but this has importance in determining causal relationships and minimizing effects due to unnaturally occurring situations. Therefore, allelopathy studies should consider more complex bioassays involving soil microbial communities, field concentrations of allelochemicals, multiple test species and using native leachate as a control that might represent the ecological phenomena in the field. For example, soil microorganisms might play an important role in influencing the bioavailability of allelochemicals in soil (Bauer et al. 2012; Ehlers 2011; Inderjit 2005), which could be achieved by the addition of microbial inoculum in experimental soil substrate collected from test species grown in the field. So, further research related to incorporation of soil microorganisms might be imperative to advance the allelopathy as one of its invasion mechanisms.

An effort has been made to overcome the concern through measuring the concentration of allelochemicals in the *P. australis* rhizosphere soil (Uddin et al. 2012), considering the osmotic potential of higher concentrations (Uddin et al. 2014a), adjusting pH, and measuring the quantity of litter biomass produced per unit of soil or covered area. These criteria in our previous studies have been taken into consideration in this current study. Moreover, separation of allelopathic effects from resource competition is a vital point in allelopathy research which has been addressed in this study, indicating phytotoxins secreted by different means from *P. australis* are responsible for invasion process except root exudations. Despite the results indicating less inhibition of root exudates by *P. australis* on *M. ericifolia* transplanted plants (Uddin et al. 2014b) but toxin may arise from other sources such as residue decomposition into soil, inhibiting germination processes and other growth parameters (Uddin et al. 2014c). These results are well aligned with other allelopathy studies of *Agropyron repens* in which Welbank (1960) found that decaying roots and rhizomes of *Agropyron* markedly inhibit the root and shoot growth of rape seedlings but no significant inhibition by root secretion. On the other hand, plant–plant allelopathic interactions may be explained by species-specific (Hierro and Callaway 2003; Prati and Bossdorf 2004) and contextual relationships (Bauer et al. 2012) that may prove the consistency of whole results of our studies. Finally, it can be said that the possibility of allelopathy as a probable cause of plant growth inhibition in some natural systems is not denied, but it has not yet been proven to be the sole factor of interference in any study. There are other possible explanations of the effect, e.g. volatilisation, mechanical root interaction etc.

## Conclusions

The overall observation of growth reductions in test plant species at low densities was inconsistent with the standard resource competition hypothesis and provides support for the hypothesis of chemical interference by *P. australis*. Although, the growth response of test species did not follow the consistency in all experiments, in most cases, the results demonstrate the density-dependent phytotoxicity concept. Therefore, these studies may provide an understanding of plant–plant allelopathy interactions and may distinguish the mechanisms involved in plant interference i.e. resource competition and allelopathy. Our findings may be useful to evaluate the response of agricultural plants such as *L. sativa* to weed residues, and may also provide insight evidence of allelopathic potential in *P. australis* invaded wetlands. In addition, the density-dependent phytotoxicity phenomenon may bring important ecological implications as a methodological approach in allelopathy (Weidenhamer and Romeo 1989).

## Additional file

**Additional file 1: Figure S1.** Effects of *Phragmites australis* litter leachate on (A) above-ground biomass and (B) below-ground biomass of *Rumex conglomeratus* seedlings at different densities. **Figure S2.** Relationship of (A) above-ground biomass and (B) below-ground biomass with seedling densities of *Lactuca sativa* grown at different concentrations of *Phragmites australis* litter (unburnt versus burnt) extract. **Figure S3.** (A) Above-ground biomass and (B) below-ground biomass in different concentrations of *Phragmites australis* litter mediated agarose at different seed densities of *Lactuca sativa*.
