# Supplementary material for: Allelopathy and resource competition: the effects of Phragmites australis invasion in plant communities
Source: Bot Stud. 2017 Jun 29;58:29. doi: 10.1186/s40529-017-0183-9 (PMC5491426; doi:10.1186/s40529-017-0183-9)
Supplement: Supplementary file 1 — Additional file 1: Figure S1. Effects of Phragmites australis litter leachate on (A) above-ground biomass and (B) below-ground biomass of Rumex conglomeratus seedlings at different densities. Figure S2. Relationship of (A) above-ground biomass and (B) below-ground biomass with seedling densities of Lactuca sativa grown at different concentrations of Phragmites australis litter (unburnt versus burnt) extract. Figure S3. (A) Above-ground biomass and (B) below-ground biomass in different concentrations of Phragmites australis litter mediated agarose at different seed densities of Lactuca sativa. [file 40529_2017_183_MOESM1_ESM.docx]

**Additional file**

**Allelopathy and Resource Competition: The Effects of *Phragmites australis* invasion in Plant Communities**

Md Nazim Uddin^1, 2, *^ and Randall William Robinson^1, 2^

^1^ Department of Ecology & Environmental Management, College of Engineering & Science, Victoria University, Vic. 8001, Australia.

^2^ Institute for Sustainability & Innovation, Victoria University, Vic. 8001, Australia.

^*^ Corresponding author. E-mail: Nazim.Uddin@vu.edu.au


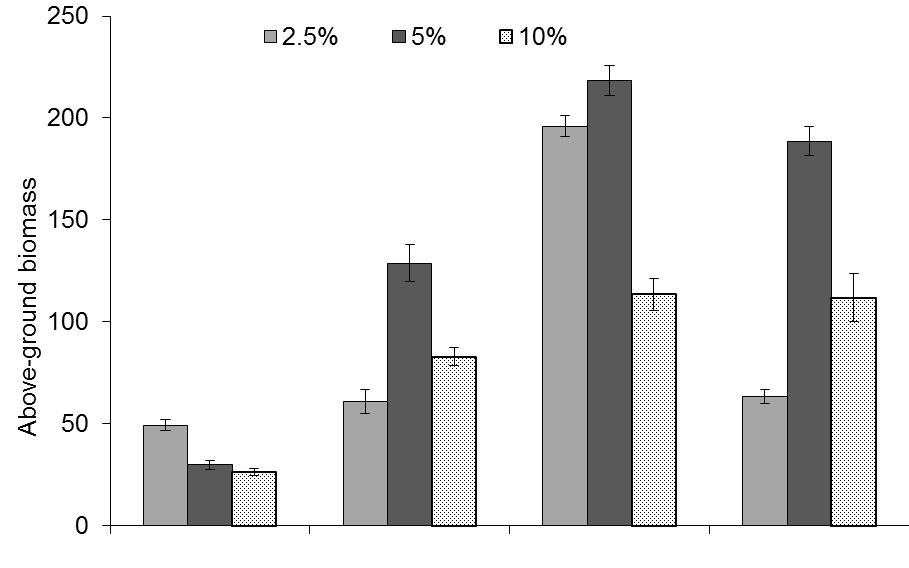


a

b

b

a

b

a

a

a

b

a

b

c

A


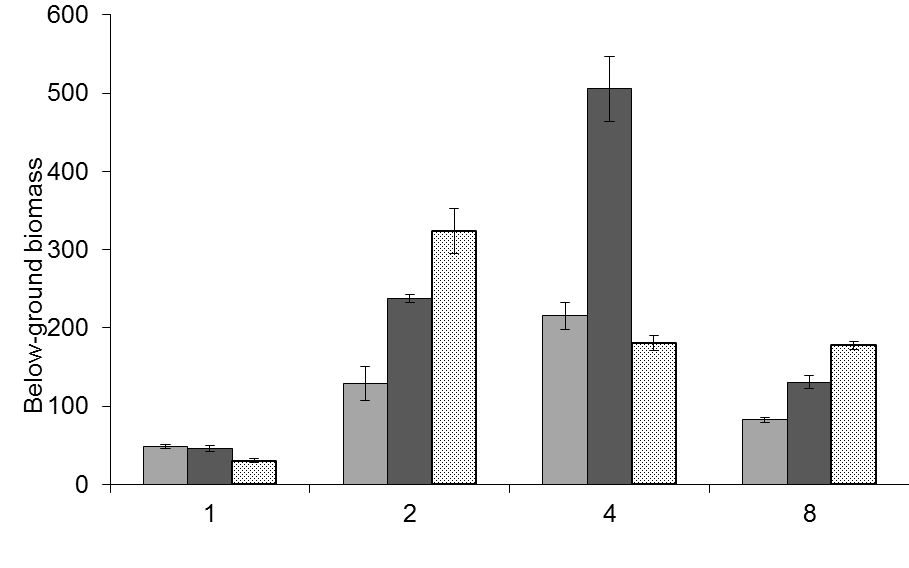


a

a

b

a

b

c

a

b

a

a

b

c

B

Plant density

**Fig. S1** Effects of *Phragmites australis* litter extract on (A) above-ground biomass and (B) below-ground biomass of *Rumex conglomeratus* seedlings at different densities. Values (weight per plant) are means as % of control treatments at each density ± standard errors (*n = 3*). Letters indicate homogenous subgroups (*P* ≤ 0.05) at each density in Duncan’s test.


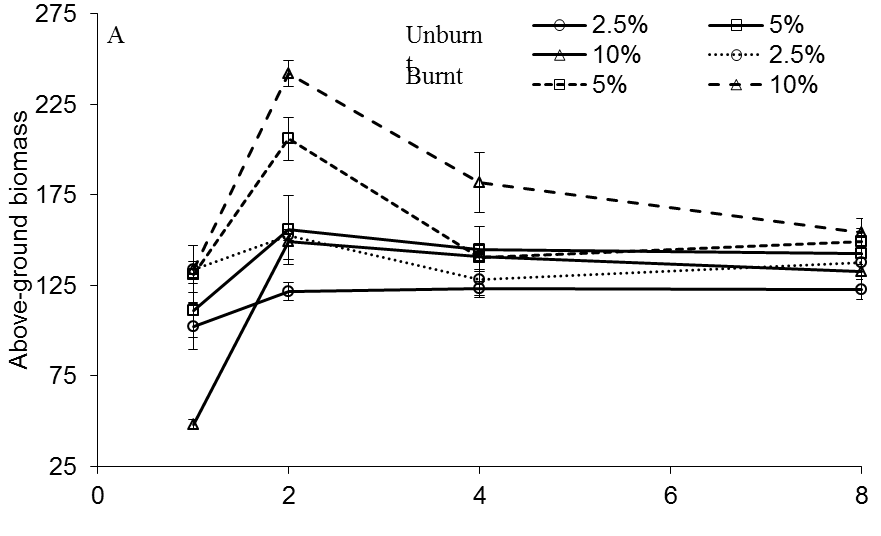

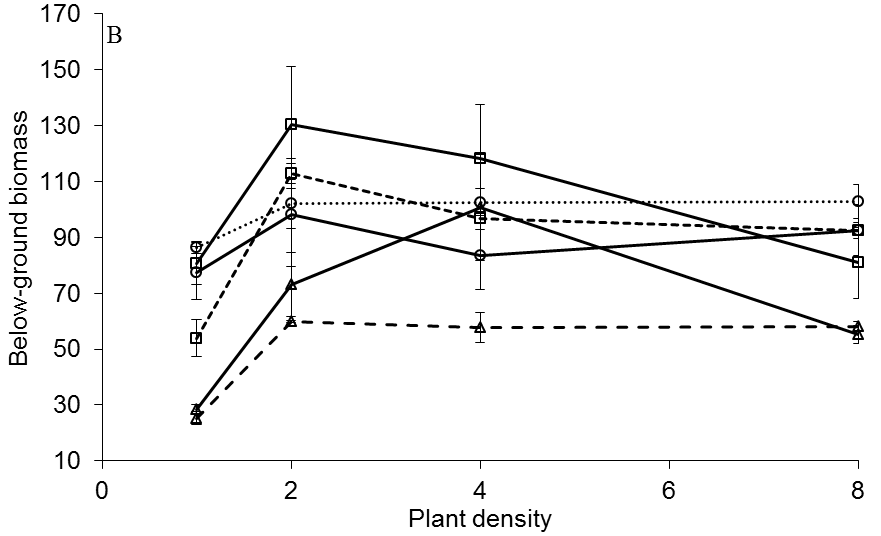


**Fig. S2** Relationship of (A) above-ground biomass and (B) below-ground biomass with seedling densities of *Lactuca sativa* grown at different concentrations of *Phragmites australis* litter (unburnt versus burnt) extract. Values (weight per plant) are means as % of control treatments at each density ± standard errors (*n = 3*).


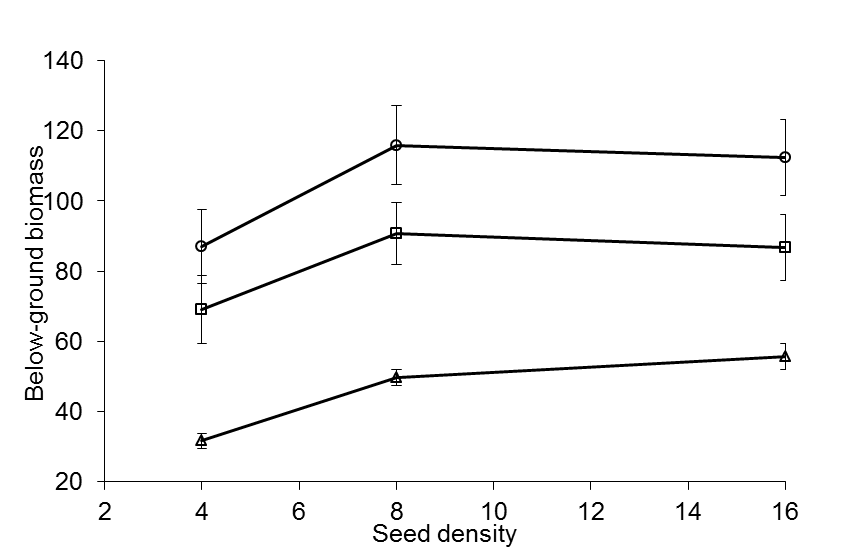


B

a

a

b

a

a

b

a

a

b


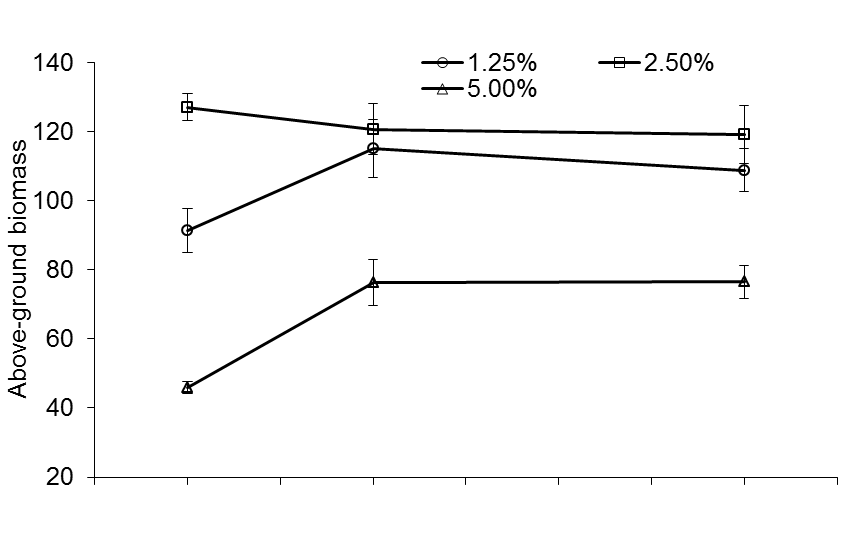


A

a

b

c

a

a

b

a

a

b

**Fig. S3** (A) above-ground biomass and (B) below-ground biomass in different concentrations of *Phragmites australis* litter mediated agarose at different seed densities of *Lactuca sativa*. Values (per plant) are means as % of control treatments at each density ± standard errors (*n = 3*). Letters indicate homogenous subgroups (*P* ≤ 0.05) at each density in Duncan’s test.
